# Dublin Hospital Reports

**Published:** 1823-09-01

**Authors:** 


### *Dublin Hospital Reports and Communications, &c. Vol.* III.

[Concluded from page 102 of this Vol.]

Our readers are aware that we concluded our first analytical article of this volume with Dr. Colles's observations on the dangers of dissection. We now proceed to analyze the remaining papers in the order of their standing in the work before us.

### 1.—*On the Application of Leeches to Internal Surfaces.* By Philip Crampton, M.D. F.R.S.

It is generally admitted, as Dr. Crampton observes, that local blood-letting and counter-irritation are among the most powerful means which art supplies for the relief of local inflammation. It is reasonable to infer that the blood should be taken as directly as possible from the tissue which is the immediate seat of the inflammation, and that the counter-irritation should be excited in a tissue different and remote from the seat of the morbid action. Yet this principle does not seem to have been very distinctly stated or rigorously acted on by systematic writers. "The application of leeches to the skin of the eye-lids, and of blisters to the temples for the relief of ophthalmia, (says our author) is clearly a violation of it, both in respect to the bleeding and the blistering; since the blood is not drawn *directly* from the tissue which is the seat of the inflammation, and the blister is applied so near to it, that the irritation which it excites, not unfrequently extends to the diseased part, where it acts as a *direct* instead of a *counter-irritant.*"

Having in many instances, observed what little impression is made upon the inflamed and turgid vessels of the conjunctiva by the application of leeches, even in great numbers, to the eye-lids or temples, and that when applied to the eye-lids in particular, they frequently excited erysipelatous affection, our author was led to try the effect of applying a single leech to the inflamed conjunctiva itself, where it covers (lines) the lower eye-lid. The result exceeded his most sanguine expectation, as a chronic inflammation of the eye which had subsisted for many weeks, was immediately relieved, and by a second application, was, in two or three days completely removed. This success led him to extend the practice to every case of inflammation of the conjunctiva, and he can now, with confidence, recommend this practice as affording the most powerful means of which we are possessed of subduing inflammation of the eye, whether chronic or acute, and whether affecting the conjunctiva or the more interior structures of the organ. The practice, he informs us, is now generally adopted in Dublin. Dr. Crampton subjoins a report of the ophthalmic cases received into the Royal Military Infirmary during the last seven years, as affording the best testimony that can be adduced of the safety and efficacy of the practice here recommended. It appears that during the abovementioned period 2074 were received, of whom 5 were blind when admitted, and 9 passed afterwards for pensions in consequence of lost or impaired vision. There went cured then to their respective regiments 2060 out of the 2074—an astonishingly large proportion considering the delicacy of the organ affected. In a large majority of these cases (including every kind and degree of inflammation of the eye) leeches were repeatedly applied to the conjunctiva, under the direction of Staff-Surgeon Stringer, and our author can distinctly state that, not only in no instance was the practice attended with any inconvenience, but that in by far the greater number of cases, the application of one, or at the utmost, of two leeches to the conjunctiva, had a more decisive effect in unloading the inflamed and turgid vessels of that membrane, than the application of five times that number to the temple and eye-lids. It is hardly necessary to observe that this measure is not to supersede the other proper measures usually resorted to in cases of ophthalmic inflammation. The present remedy is evidently one of considerable economy.

"With respect to the manner of applying leeches to the conjunctiva, nothing can be more simple. The patient should be placed with his back to the light, in order that the lower eye-lid may be everted without exciting pain; a leech or two, rather below than above the middle size, should be allowed to fix upon that part of the inflamed membrane, which covers the tarsus, taking care that it fastens neither upon the ciliary margin, nor upon the eye itself.* The leech fixes and fills himself in this situation much more quickly than upon a cuticular surface, and this observation is equally true with respect to all internal surfaces, for which I observed that leeches have the strongest appetency.†

Encouraged by this success our author was led to extend the principle to a surface more difficult of access than the conjunctiva—viz. to the mucous membrane of the fauces or throat, in severe cases of cynanche tonsillaris. He first made the attempt in the case of a young person who had a severe inflammation of this kind, and it was effected with less difficulty than was expected.

"A single thread of silk was passed *through* the body of the leech, at about its lower third, the ligature being made fast to the finger of the operator, the leech (held securely between the fore-finger and thumb) was introduced into the mouth, and its head, directed by a probe, was brought in contact with the inflamed tonsil. The animal fixed itself to the part in an instant, and, in less than five minutes, being gorged with blood, it fell upon the tongue, and was withdrawn. The relief was immediate. The tonsil continued to bleed for three or four hours. On the following day the inflammation had greatly abated, and in a few days the tumor had completely subsided without suppuration." 229.

In the month of September, 1819, our author was called to a gentleman who had been suffering for 16 hours under a severe attack of cynanche tonsillaris, the tonsils being in contact from the inflammation, which had extended to the velum and uvula, the latter being œdematous. Two leeches were directed to be applied to the tonsil and velum, and an emetic to be taken after the leeches fell off. The leeches procured immediate relief, and on the day following, the patient was able to leave his room. He could report many similar cases, but thinks the foregoing sufficient. When scarlatina was prevalent in Dublin two years ago, he had numerous opportunities of witnessing the great relief obtained by the application of leeches to the tonsils, when the inflammation of the mucous membrane ran high, and he can, therefore, state with

* "In several instances I have, (from inattention upon the part of the operator,) seen the leech fix upon the conjunctiva which covers the sclerotica, but this was attended with no other inconvenience than a temporary ecchymosis."

† "In Egypt and in the Balearic islands those who drink of the stagnant waters are subject to great inconvenience and even danger, from a small leech, which attaching itself to the internal fauces, continues to suck blood, until, from the size of a horse hair, it acquires the dimensions of a common leech." 228.

confidence, that local blood-letting, in this way, when early employed, affords one of the most powerful means of relieving the most distressing symptoms of this dangerous disease.

The application of leeches to the nostrils, vagina, and other internal parts, so common on the Continent, has not yet received such notice in this country as it deserves. Dr. Crampton's paper will, in all probability, excite the attention of our brethren to the subject.*

2.—*Case of a large Coagulum of Blood and Urine extracted from the Bladder, by means of a Syringe.* By L. Byron, M.D.

It is a well known fact, that extravasated blood will coagulate at the ordinary temperature of the human body, so that when effused into the bladder, and mixed with the urine, in cases of injury or disease, one firm mass of coagulum may be formed. To a moderate extent this is not of much consequence, as it will be gradually dissolved and removed; but should the bladder be so far distended with blood as to have its office obstructed, or the function of the kidneys affected, the case becomes one of great importance, and requires the promptest measures for relief.

In the following case much benefit was obtained by the use of the syringe, although the application of the instrument was too long delayed to effect a cure.

On the 9th of November, 1820, a robust man, 64 years of age, was attacked with retention of urine, which he ascribed to hard riding during the preceding day, the air being damp and cold. Our author saw him on the evening of the 10th, 30 hours after the attack had commenced. The pain (referred to the glans penis) was excruciating, with constant desire to empty the bladder, and frequent stools with tenesmus. Three pints of high-coloured urine were drawn off by the catheter, considerable resistance being given to the introduction of the catheter by a stricture near the bulb of the urethra, and also by an enlarged prostate gland. Twenty ounces of blood were taken from the arm—the bowels were freely opened by castor oil—the warm bath was used—leeches applied to the peri-

* Since the foregoing analysis was drawn up, an anonymous writer in the Medical Repository for June last, confirms the report of Dr. Crampton, as being an eye witness of the success of the measure recommended. He quotes a passage from Magneto, a Prussian physician, in the year 1703, which hints at the application of leeches to internal surfaces; but only the internal nares are mentioned in the passage of Magneto.

næum—an enema exhibited—and fomentations applied to the hypogastrium and perineum. In six hours after catheterism the symptoms became so urgent as to render a repetition of it necessary, by which 20 ounces of high-coloured urine were drawn off with much relief. The urine was obliged to be drawn off every day till the 15th, when, having made a little water while in the bath, he found himself so much better as to sit up for three hours, which imprudence was followed by an aggravated degree of all his former sufferings. When the catheter was introduced (with much difficulty) nothing passed but a few drops of blood. By management of the instrument, however, four ounces of urine were abstracted, followed by blood which obstructed the openings of the catheter, and rendered its removal necessary. The antiphlogistic means were rigorously used for two days, without benefit—and no urine had passed during that time. The bladder could be felt distended through the rectum—the pulse was 100°—tongue loaded—countenance expressive of much anxiety—hiccup. On the 18th, a large sized silver catheter, with one oval opening near the extremity was introduced into the bladder, and the pipe of an eight ounce syringe was adapted to the external orifice of the instrument. By repeated quick strokes of the piston, two ounces of firm coagulum of a black colour and urinous smell were drawn off; and by repeating the operation sixteen ounces more were abstracted, affording great relief to the patient. 19th—About eight ounces of a ropy dark brown matter were drawn off. 20th—Six ounces abstracted. 21st—Some ropy urinous matter passed involuntarily. He expired on the 24th in a state of coma, the catheter having been introduced the preceding day, but the bladder found empty. No leave was permitted to examine the body.

Our author considers it remarkable that surgical writers should be altogether silent on the use of the syringe in cases of retention of urine arising from coagulated blood in the bladder. This case clearly proves that the syringe has power over such coagula, and we have no doubt that it will be used on proper occasions by our surgical brethren.

### 3.—*Case of Obstructed Œsophagus, &c.* By John Howship, Esq.

Mr. Howship was summoned to an aged woman in one of the infirm wards of the St. George's Infirmary, who was said to be dying. He found her on her feet, with every expression of despair in her countenance, oppressed by the most extreme dyspnœa, and on the eve of suffocation. He was told something had stuck in her throat while eating, and on enquiring where she felt distress she pointed to a particular part of her throat, which appeared to be opposite the upper edge of the sternum. Having patched up a temporary probang, (it is rather surprising that there should not have been such an instrument in the Infirmary) of such implements as he could procure, Mr. Howship passed it down the throat of the woman to the piece of meat forming the obstruction, and pushed this substance down nearly into the stomach, when relief was immediately obtained, it being evident that the mechanical pressure on the trachea had been the sole cause of the severe symptoms.

4.—*On a Paralytic Affection of the Superior Extremity.* By Robert Healy, M. B.

This affection, our author has not seen described in books, and believes it to be unconnected with any organic disease of the brain or nervous system. It attacks persons of all ages and both sexes. Prior to the assault the patient, generally speaking, is in the enjoyment of good health, consequently is much surprised to discover, on waking from a sound sleep, a total loss of power of one hand, and what is remarkable, the loss of power is decribed as extending to the middle of the fore-arm—in some cases to the elbow, accompanied with a sense of great numbness. No other cause could be assigned by the patients than the probability of pressure on the nerves occasioned by lying with the head resting on the arm. Most of the usual remedies for palsy had been found ineffectual; but the disease invariably yielded to electricity strongly applied so as to draw pungent sparks from the diseased arm or hand.

5.—*A Case of Perforation of the Perineum.* By John C. Douglass, M.D.

Every one is acquainted with the common lacerations of the perineum which occasionally take place during the expulsion of the fœtus, even when the greatest care is employed. Indeed we are among those who believe that "supporting the perineum" as it is called, has little effect in preventing the accident, which occurs as often when the hand is applied as where there is no such artificial support. But be that as it may, the accident here described is of a more rare occurrence, and of a different kind. It has been termed by Denman a perforation or bursting of the perineum, and, according to Dr. Douglass, instead of being in the centre, it is usually situated towards one side of the perineum, involving in its necessary amplification the labium pudendi of the same side, with other parts, as will be shewn in the following case:—

A woman, aged 23, in her first labour, was in the lying-in-hospital on the 24th January, 1810. She was attended by a raw young pupil, and notwithstanding the previous tranquillity, the pupil and nurse were surprized and alarmed to hear the woman utter a shriek expressive of unusual suffering. Dr. Douglass was therefore quickly summoned, and found that the head of the child had protruded—but instead of being in the usual position, it was closely applied to the inner and posterior part of the patient's left thigh. While reflecting on these circumstances, another pain protruded the child's body. On viewing the parts, a shocking laceration was discovered, having the appearance of a large wound inflicted by external violence. Although denominated a perforation of the perineum, yet the opening was only partly comprised in the perineum laterally, partly in the left labium pudendi, but chiefly in the integuments of the thigh. Our author found that the placenta obstinately followed the course which the fœtus had pursued.

Notwithstanding this extensive injury, no extraordinary activity of after-treatment was rendered necessary to obviate febrile action, and the woman ultimately recovered by judicious local treatment of the wound. Dr. Douglass is of opinion that, in this case, the head of the fœtus bearing upon the cervix uteri (the os uteri perhaps not sufficiently dilated) burst through the former, and through the vagina, passing completely behind the latter, at or near to the sacro-iliac synchondrosis.

### 6.—*Cases of Jaundice, with Dissections.* By Henry Marsh, A.B. M.D.

Jaundice, as our author observes, is produced by several different causes. In the majority of instances, however, its origin may be traced to some obstruction of a mechanical nature, as gall-stones, tumours, indurations of the liver, preventing the free egress of bile from the proper organ to the duodenum. These cases have been amply described and discussed by writers of all kinds; but there certainly are many cases of jaundice in which these mechanical obstructions are not apparent after death. It is still a question at the same time, whether or not some actual obstruction did take place in the biliary ducts during life, occasioning the suffusion of bile on the surface. That spasmodic constrictions of the bile tubes do sometimes take place we have had *post-mortem* evidence; and that the same should take place during life, and yet become effaced by death, we cannot rationally doubt, from analogy and fair reasoning.

The object of this paper is to bring forward instances in which icterus is connected with derangements of other and distant parts, as the brain and mucous membrane of the intestines. The first case which arrested our author's attention was one already published in the first volume of the Dublin Hospital Reports, by Dr. Cheyne, from Dr. Marsh's notes. The subject was a wretched young woman, broken down by disease and mercurial courses in the Lock Hospital of Dublin. She was seized with phrenitis, (while labouring under jaundice,) and died quickly.

"The condition of the large intestines in this female was very remarkable; the quantity of knotted fæces which occupied the intestinal pouches was almost incredible; and this their condition the more claims attention, when it is known that alvine evacuations had been regularly maintained during the whole time of the residence of this patient in the hospital. These were fluid and watery stools; such as are frequently carried off by medicine, whilst scybala in abundance remain behind. That this is often the case every physician is aware: but I believe it is not generally known that this state of the large intestines is sometimes a cause of jaundice.* While pursuing the dissection of the body of this female, the following curious appearance was exhibited: bile, mixed with a substance resembling curd, flowed in considerable quantity from the lactiferous ducts; the mammæ appeared full, and by a very moderate pressure there were obtained several ounces of a tenacious yellow substance, bearing all the visible characters of pure bile; all the serous membranes, as well as the skin and adnata, exhibited likewise the bilious tinge." 263.

We cannot certainly admit that bile or any other secretion can appear in the circulation anterior to its elaboration in its proper organ; but we have strong proofs that a fluid, when once secreted and obstructed in its natural outlet, may become absorbed; as urine, for instance, has been deposited in the ventricles of the brain, or thrown off by the skin or stomach. Of the latter fact our author has lately met with a well attested instance. In like manner the bile having been secreted, and prevented egress by the biliary duct, is absorbed, and appears in the skin, urine, tears, serous membranes, and brain. Our author received the following communication from Dr. Cheyne lately.

" 'Last summer I visited a lady in jaundice with the following

* "Of this fact I was then ignorant, nor was it until long afterwards, having met with a similar case, that I was led to the conclusion, that a long continued obstruction in the large intestines becomes occasionally the immediate cause of icterus. The symptoms of the case which led me to adopt this opinion I shall presently relate."

symptoms:—Skin and urine deeply charged with bile; slight anorexia; languor; stools pretty natural, as were the tongue and pulse. The indisposition was so slight that the individual in question had no intention of sending for a physician, till she discovered that the bilious tinge of her skin was imparted to her linen. To satisfy my doubts, she repeatedly wiped her face with a cambric handkerchief, which thereby acquired a saffron colour. Her liver was not sensibly affected in respect of enlargement or tenderness. She speedily recovered; indeed her recovery might be termed spontaneous, nothing having been prescribed for her but some mild purgative medicine. She was directed to use light animal food, to abstain from wine, and avoid fatigue." 269.

We shall now proceed to notice the case which our author has brought forward in illustration of jaundice from causes situated in the large intestines—the sympathetic connexion subsisting between the functions of the skin and liver being also remarkably exemplified in the same case.

An Octogenarian, of very irritable and anxious temper, long troubled with scrotal hernia and hydrocele, became affected with jaundice, for which he was treated by Drs. Perceval and Falloon, and the disease was removed. Several months afterwards our author was suddenly summoned to the old gentleman, in consequence of a discharge of blood from the rectum. He had complained of a dull heavy pain in that region for some time previously, especially when at stool. When seen the skin was yellow, harsh, and dry—the adnata deeply tinged—the pulse full, intermitting, but not quickened—the tongue black, dry, and thickly coated—urine scanty and high-coloured—vertigo—incoherence—somnolency. He was sitting on the night-chair much alarmed. The matter evacuated appeared to consist of pure, dark-coloured, clotted blood; and there were at the bottom of the vessel some hard round fæces. Suspecting the cause both of the hæmorrhage and icterus, our author ordered him two grains of rhubarb, the same of scammony, and the same of sulphate of potash, every two hours, till the bowels should be well cleared. By the following day a large vessel had been more than half filled with scybalæ, which were tinged with blood. A continuation of the same treatment removed large quantities of these hardened fæces, and with them the jaundice and all the other bad symptoms.

Whenever these accumulations were re-produced, the jaundice re-appeared, and with it loss of memory, drowsiness, and incoherence. On one occasion, some slight paralytic affections accompanied the other phenomena, and were removed with them by the evacuating medicine abovementioned. By proper diet and aperient remedies the health was restored, as far as could be expected at his advanced age.

"In this case," says our author, "we are furnished with another instance of the connexion which exists between obstruction in the large intestines and jaundice; and also of the connexion which may be traced between this latter symptom, and an excited and deranged condition of the cerebral functions. Here too, as in the former case, aperient medicines were daily administered; fluid fæces, slightly tinged with bile, regularly evacuated; and yet the bowels remained loaded with solid matter. I have remarked, in dissection, that in those pouches in which scybala are lodged, the mucous surface, against which they rest, is frequently reddened and vascular. The irritation of these hard substances accounts for the hæmorrhage, which in this old gentleman had been attributed to internal piles: it seems to me also that the loss of blood added much to the efficacy of the purgative pills, which probably would not otherwise have so effectually unloaded the bowels." 273.

We are disposed to think that the obstruction to the free course of the bile, in the foregoing and in analogous cases, is mechanically caused by impacted fæces in the colon, pressing upon the ducts of the liver.

Our author lays it down as a good rule, for the propriety of which we can vouch, that old people ought to be very abstemious if they wish to preserve a clear intellect. In old age the digestive, like all the other functions, become weak, and exercise, so necessary for due action in the chylopoietic viscera, is almost annihilated. Hence the necessity of proportioning the ingesta to the digestion and exercise.

A strong muscular man, 40 years of age, was admitted into one of the fever wards of the House of Industry, during the prevalence of the epidemic fever, deeply jaundiced—tongue perfectly dry and nearly black—pulse natural. He had a wildness in his countenance—declared he was quite well—and wished to quit the hospital. Arteriotomy and a purgative were ordered, but he obstinately refused all medicines, soon became furiously delirious, and died in convulsions the same night. Permission was not granted to open the body; but our author thinks that this is another instance of jaundice, terminating fatally in cerebral and nervous affection.

A young girl in the Lock Hospital, who had got cold while under the influence of mercury, was suddenly told of the death of an uncle, the only man who had shewn her any kindness. She immediately became universally jaundiced, and soon afterwards febrile symptoms appeared. She was sent home, and continued feverish for some days, as also jaundiced. At length quite suddenly she became affected with alarming symptoms, as stertorous breathing, partial insensibility, great pain in the abdomen. As night approached she could hardly be restrained in bed—she screamed violently, and was constantly agitated. At 12 o'clock of the succeeding night she expired in convulsions. On dissection, which was performed a few hours after her death, some congestion was found in the vessels of the brain, but without any effusion. The substance of the liver was deeply tinged with bile, its structure being unaltered. The gall-bladder was contracted, containing some dark green bile—a probe could not be passed through its duct—the other ducts unobstructed. Around the orifice of the ductus communis in the duodenum the mucous membrane was highly vascular—the fluid in the duodenum was untinged with bile. There was some increase of vascularity in the mucous membrane of the stomach—small intestines remarkably contracted in several places. "This," says our author, "is another decisive instance, wherein jaundice, caused by strong mental emotion terminated in a violent and fatal affection of the brain."

Dr. Colles related to our author a somewhat similar case that fell under his care. A young gentleman, after going through an alterative course of mercury for the cure of a chancre, suddenly became deeply jaundiced, in three days after which he was seized with delirium, followed by repeated convulsions, which terminated in death. The most accurate examination of the body did not disclose any appreciable change of structure. The external and internal parts were much tinged with bile.

Our author quotes some analogous cases from Morgagni, and then proceeds to make some reflections. He properly remarks, that although in ordinary cases of jaundice there appears little or no cerebral disorder, yet that under certain circumstances, and in certain conditions of the nervous system phrensy may be excited, either by the bile conveyed to the brain, or in consequence of the sympathy which exists between the cerebral and biliary systems. In practice, therefore, we should be aware that an icteric patient, who has a weak and irritable nervous system, must be closely looked after, lest alarming symptoms unexpectedly arise, and lest we should be unguarded in our prognosis. The reciprocal influence of the brain on the liver, and of the liver on the brain, has long been observed and acknowledged. The following case illustrative of the fact that, in affections of the brain, the occurrence of jaundice indicates the rapid approach of dissolution, is very interesting in many points of view.

A gentleman, 36 years of age, muscular and well formed, had been intemperate in his youth, and had undergone several courses of mercury for syphilitic complaints—was habitually subject to head-aches, especially after any excess in drinking. He was bilious, nervous, and irritable.

On the 23d of April, he had a fall on his side from a scaffold, about eight feet high, and was slightly stunned, but apparently not injured. In the course of the evening he complained of confusion in his head, which continued more or less till the 2d of May, when he had slight rigors and general indisposition. On the 4th he dined with a friend in Dublin and drank moderately. Next day he rode out into the country, where he was suddenly seized with violent pain in his head darting from temple to temple, and from the occiput down to the sacrum. On the 6th, there was an entire remission of the pain, but it returned at night. 7th, the same remission and exacerbation. 8th, was leeched plentifully, and purged freely. No relief ensued, and he was sleepless. On the 9th our author first saw him. The pain was in his temples and forehead—he appeared much agitated—complained of total sleeplessness—sighed occasionally—moaned often—pulse natural—skin cool—bowels free—tongue very white and moist. In the evening the pain became so intense that he requested to be bled. As his pulse had risen in strength and frequency, and as he was in constant agitation, a vein was opened, and he was allowed to bleed till he fainted, which produced considerable relief. The pain returned, however, shortly afterwards, upon trifling exertion, and with it the restlessness. He took twenty drops of laudanum, and slept two hours, when he awoke complaining of much pain, his countenance and manner expressive of unusual wildness. His pulse was full and hard, not frequent—skin hot. He was again bled, but syncope threatened before eight ounces of blood were taken. He was materially relieved, and passed a tranquil though sleepless night.

"10th. Symptoms very alarming; pain intense, seated in the forehead, temples, and at both sides of the head, increased by the least exertion, even by the effort of speaking. When unexpectedly addressed, or touched with any cold substance, he was thrown into a state of great agitation: he complained of intolerable pain; could not breathe freely; a sort of convulsive struggle or catch affected the respiratory organs; his sighs were deep and frequent. Pulse full, and hard, and 92 in a minute; tongue loaded with a thick white secretion. Under these circumstances, bleeding was again resorted to. Twenty ounces were quickly removed; there was but a slight tendency to syncope; the symptoms were materially mitigated, but by no means subdued. At ten at night he was nearly in the same state as in the morning. In his extreme restlessness, he removed the bandage from his arm, and bled copiously. The relief was instantaneous. He passed a quiet though sleepless night. Ice was applied to his forehead, and every fourth hour a cup of the following mixture given:—℞. *Aq. ferv.* ℥xii. *Crys. Tart.* ʒi. *Ant. Tart. gr.* i. *Sac. albi* ℥ss. ♏." 286.

11th. He appeared better, but there was an evening exacerbation. 12th. Still better; but at midnight an exacerbation of pain with restlessness. Sixteen leeches to the head, which brought great relief. Heaviness and numbness in the head were now the only symptoms complained of; but he could not sleep. 13th. Unceasing loquacity, peevishness, and irritability—at night his intellect deranged for the first time. 14th. Severe pain in the right temple. Ten leeches applied, which entirely removed the pain, but rendered him very faint. Now came on muttering and incoherence, grinding of the teeth, heaviness about the eyes, impaired memory. His tongue was clean, skin cool, pulse natural. 15th. Stupor—remarkable change of countenance, being of a deep yellow tinge—night sleepless. 16th. Took food with pleasure—upper half of the body covered with a profuse perspiration of a peculiar and heavy smell—seized with convulsions, and expired at 4 P. M.

" Dissection,—Performed twenty-four hours after death. On raising the dura mater, a thin layer of coagulated blood, deposited between the arachnoid membrane and pia mater, extended on each side over a considerable space of the anterior lobes of the brain. A similar appearance was observed at the superior and posterior parts of the brain, under the parietal bones, and reaching downward to the occipital. Blood was likewise effused between the same membranes at the base of the cranium, and over the surface of the cerebellum; a layer of blood was also deposited between the convolutions in every part of the brain. The lateral ventricles with their cornua, the third and fourth ventricles, were entirely filled with one large, continuous clot of dark blood: connected with this, another, as large as a walnut, occupied the inferior part of the left anterior lobe, into which a ruptured vessel, capable of admitting a small injecting pipe, opened. The quantity of coagulated blood in the interior of the brain amounted to several ounces: we were allowed to examine only the head." 289.

There is, Dr. Marsh observes, a form of jaundice which appears in hysterical women, owing its origin, probably, to some commotion and disturbance of mind. The symptoms, at first, would lead to the idea that the patient laboured under a violent attack of bilious colic, or that a gall-stone was impacted in the ducts. Sydenham has given a description of this disease, as attacking women of a lax and gross habit of body, and such as have laboured under an hysteric affection, or been much reduced by difficult labours. "A pain, says he, not less severe than that of the iliac passion, is felt at the region of the stomach, or somewhat lower, which is succeeded by copious vomitings of matter, sometimes green, sometimes yellow. To these symptoms are added a depression of mind, and despair exceeding that in any other disease. After a day or two the pain subsides, but returns with unabated violence in a few weeks. It is accompanied sometimes with a remarkable jaundice, which spontaneously subsides in a few days. The least commotion of the mind, whether it be anger or fear, brings back the pain." *Sydenham.*

"In the cases," says Dr. Marsh, "of this complaint which I have seen, serious mischief was done by mistaking the attack, in one instance for colic, in another for gall-stones. Where hysteria is at all suspected, the urine should regularly be observed: its limpid and copious secretion will often at once detect the real nature of the affection, and prevent all the injury resulting from needlessly active and vigorous treatment. The hysteric colic and jaundice are easily subdued. A few leeches, fomentations, moderate purgation, followed by opiates and tepid baths, will relieve the urgent symptoms; and in the cases which have fallen under my observation, change of scene, regular and wholesome diet, exercise on horseback, and mild aperients, have prevented their return. In these cases the subsiding of the pain was always accompanied with extrication and discharge of flatus, either upward or downward. It is singular that Heberden, so accurate an observer, should deny that there is such a disease as jaundice connected with hysteria, and blame Sydenham for having supposed its existence. By him the symptoms are referred to gall-stones:—indeed, in what he has written on the subject, he seems hardly to contemplate any other cause of Icterus." 291.

Although we do not agree with Heberden in supposing that all these cases depend on gall-stones, yet we firmly believe that either a spasmodic constriction of the gall-ducts, or an obstruction of them by viscid bile exists at the time, and forms the leading proximate cause of the whole phenomena.

Our author next proceeds to the connexion subsisting between a jaundiced appearance of the skin, and a morbid condition of the mucous membrane of the bowels.

Here, as in the form just described, dissection leaves us in doubt respecting the immediate cause of the jaundice. The affection of the mucous surface of the bowels, our author observes, is constantly overlooked. Its symptoms are often obscure, and it may, he thinks, exist to a considerable extent, yet leave no trace of such existence after death. This is not wonderful when we consider that a recent inflammation on the external skin seldom retains in the dead body any evidence of its previous existence. In such delicate structures also as the meninges of the brain, violent excitement may go on during life, without leaving cognizable traces on dissection.

"The usual cause of that form of jaundice, the consideration of which will occupy the remaining pages of this paper, is the ingurgitation of cold fluids into the stomach, when the body is much heated; or sudden and reiterated exposure of the person after severe exercise, to cold. In all such cases, the disease is not confined to the liver and its ducts; but the mucous surfaces of some part, or all the intestinal canal, are involved in the same morbid action. It is even probable, that, in these surfaces, disease first establishes itself, for the patient is usually ailing for many days before jaundice appears; and the symptoms of which he complains are amongst the number of those, which indicate commencing disordered action in some part of the villous coat of the bowels." 294.

Our author minutely details a long case of jaundice terminating in dysentery, of which we can only give the more prominent features.

A labourer, ætat. 55, strong and muscular, had been thrown into a profuse perspiration by severe labour, and in this state drank repeated draughts of cold milk. During the next and several succeeding days, he felt cold, chilly, and weak, and presently became much jaundiced. His appetite was now gone, and his thirst insatiable. In a fortnight after the drinking of cold liquids, our author saw him for the first time, and noted the following phenomena:—viz. occasional syncope—prostration of strength—privation of sleep—dimness of vision—depression of spirits—palpitation—dyspnœa and cough—constriction at the epigastrium with great tenderness there—nausea on drinking cold fluids—great thirst—tongue moist and white in the middle—skin and eyes of a lemon colour—urine without sediment, but tinging linen yellow—fæces white—pulse 60, soft and full—frequent and free evacuations from neutral salts, followed by considerable abatement of the symptoms. This was on the 24th July. On the 29th, sixteen leeches were applied to the epigastrium in consequence of much pain there, and then mercurial ointment rubbed on the part, a drachm every day—sulphate of magnesia was ordered to keep the bowels open. These produced much relief. On the 1st August, the appetite was craving—the thirst abated—skin lighter—urine very bilious—skin moist—great debility. 2d August, mercurial fetor—costiveness—slow pulse—purgatives operated. 4th—pain in the temples and forehead—stools darker. 6th—Occasional rigors—white fæces—pain in epigastrio—loss of apetite—periodical pains in the head. Blood taken from the arm—leeches to epigastrium—diminution of the urine—pain in the lower belly, which was removed by an enema and warm fomentations.

12th—Stools still destitute of bile—eyes and skin deeply tinged—thirst urgent—great flatulency—much comfort from the tepid bath. September 3—Stools still clay-coloured, and now tinged with blood, becoming very frequent, with pain and tenesmus. Leeches to the abdomen—warm fomentations —a blister—repeated doses of Dover's powder. The dysenteric symptoms having disappeared, he continued for some days in an improving state apparently, when rather suddenly, towards the end of September, dysentery returned with extreme violence, the stools being dark-coloured, fetid, and full of coagulated blood, the jaundice being still unabated. In this state of suffering he lingered till the 8th October, when he expired. We shall give the dissection in the words of our author.

"The following morning, at nine o'clock, the examination of the body took place. The body was very much emaciated: a deeply yellow tinge pervaded the whole surface. The peritoneum, in every part, was coloured with bile: in its cavity there was a deposition of yellow fluid, in which flakes of lymph floated; the liver was shrunk, and retracted considerably within the margin of the ribs; the stomach was distended, and presented externally a very vascular appearance. The peritoneal coat of the large and small intestines was in extensive patches, vascular, opaque, and thickened. The vessels of the mesentery crowded together, and dilated. The cardiac portion of the internal surface of the stomach presented no unusual appearance; on approaching the pyloric extremity, the inner membrane was highly vascular, and a large patch was quite black. On laying open the duodenum, its surface presented a still more vascular appearance. This vascularity was extended along the whole tract of the small intestines, and was particularly evident upon the surface of the Valvulæ conniventes. The contents of the small intestines were every where mixed with bile. The ileum, near the caput coli, presented a highly inflamed appearance, and its contents were of a chocolate colour. The whole internal surface of the caput coli was deeply ulcerated, and covered with purulent matter; its coats were thickened: this gut was full of black, foul, and rather solid fæces, mixed with clots of pure blood. The colon and rectum were ulcerated and thickened in numerous patches throughout their whole extent. Wherever the ulcerations were most extensive, there the contained matter was most solid, foul and black; clots of black blood were every where mixed with the contents of the large intestines. The liver was diminished in size; fleshy excrescences grew from its convex surface; to the touch it was remarkably flabby and inelastic; its vessels very much dilated; its colour at every section intensely yellow. The gall-bladder contained several ounces of dark green bile; its duct at the superior part was contracted to a very small size. The remaining portion of the cystic duct was considerably dilated; as were the hepatic and common ducts, which were unobstructed. Wherever the liver was cut, green bile flowed from the enlarged ramifications of the hepatic ducts. The spleen was shrivelled, and possessed less tenacity than the crassamentum of healthy blood. The kidneys were flaccid and small, and all their vessels dilated. The viscera of the thorax were carefully examined, and did not present the least morbid appearance. The dura mater was yellow; some (but very little) fluid in the ventricles; the brain remarkably firm and healthy. Of the contents of the cranium the dura mater alone was discoloured." 299.

We have invariably remarked that the *function* of biliary secretion was disordered in dysentery; and the above case offers no exception to the general rule. The liver, in fact, was organically affected; and that its function was greatly deranged appears, even by dissection, as the passage which we have marked in italics proves. We conceive also, that the hepatic affection long preceded the gastro-intestinal irritation and inflammation—and that the depraved biliary secretion was a principal cause of the latter disorder. The deeply florid tongue, the unquenchable thirst, the extinguished or canine appetite, the prostration of strength, the rapid emaciation, and, lastly, the dysentery, were all symptoms of the gastro-intestinal phlogosis. The probable immediate cause of the icteric suffusion in this case, was (thinks our author) an inflammation of the lining membrane of the ducts, whereby they were thrown into a state of spasmodic contraction. This reasoning is illustrated by a case and dissection extracted from Dr. Hunter's Treatise on Army Diseases. A phthisical patient became affected with jaundice a few days before his death, and on dissection there were found marks of superficial inflammation all over the liver. The gall-bladder was full, but no bile could be squeezed out of it. Bile of a thick and ropy consistence was found stagnating in the ductus communis, as also in the hepatic ducts. Dr. Hunter asks—"*did the inflammation in the neighbourhood of the ducts and perhaps extending to them, excite such contractions in them as obstructed the bile?*" We think it highly probable; and, indeed, that in most cases of bilious fever the icteric suffusion is caused in this way.

"If the opinion be correct, that this form of jaundice (I mean that arising from cold applied to the heated body) is generally, or perhaps uniformly, complicated with an highly excited and sometimes inflamed state of the mucous membrane of the bowels, it must follow of necessity, that the plan of cure should be directed, not so much to the condition of the liver, as to that of the neighbouring viscera. In all cases of what may be termed recent or inflammatory jaundice, in which, combined with the other symptoms, there is tenderness and fulness at the epigastrium, the treatment should be primarily and essentially antiphlogistic: bleeding, general and local; saline purgatives largely diluted; general and local tepid baths, should uniformly precede the use of mercury. I have seen cases of this kind, wherein the mercurial treatment had aggravated every symptom, yield at once to a bleeding from the arm, followed by the application of a few leeches. This I have observed, even where the pulse was not accelerated." 303.

Here our author introduces several additional cases of this form of jaundice, some of which were accompanied with purpura, on which, and several other diseases, Dr. Marsh makes many judicious remarks and ingenious comments. The cholera morbus, for instance, engages Dr. M's. attention; and he very properly observes that, as it chiefly prevails during the autumnal season, when the alterations of heat and cold are sudden and frequent—and as it is often produced by drinking cold liquids when the body is heated, so "it owes its origin less frequently than is imagined to the nature of the food taken into the stomach." The symptoms, he thinks, sufficiently indicate the presence of morbid action in the mucous coat of the bowels.

"In this disease, the functions of the liver are so much deranged, that all the symptoms have been traced by the older writers to the irritating effects of a redundant secretion of acrid bile. That the hepatic functions are greatly deranged, is certain; but that the symptoms are caused by the action of the bile upon the mucous surfaces, is now, I believe, acknowledged to be an erroneous opinion. In treating cholera morbus, the supposition that bile is the cause of all the mischief, has led to the adoption of remedies ill calculated to check the progress of this formidable malady. The patients have been deluged with diluting drinks; and even emetics and cathartics have not been withheld. When it is considered that there is a cold stage, antecedent to that of re-action and excitement; and that the vomiting and purging exist for hours before bile appears in the matter ejected;—it must be evident that there is an highly excited state of the mucous surfaces, wholly independent of the biliary secretion. The cause of the cholera morbus, the symptoms, and the appearances after death, evince that the disordered action affects both the intestinal mucous surfaces, and the liver." 313.

Our readers are aware that we have long held this doctrine, and Dr. Marsh has liberally given us credit for priority upon this subject.

There is yet another form of disease, remarks Dr. Marsh, the seat of which is in the villous coat of the stomach and small intestines—this is the infantile remittent, or, as it has been vulgarly called, "the worm fever" of children. Its characteristic symptoms, he observes, if closely analysed, will be found to point to the mucous surface as the original seat of morbid action.

"Irritation at the extremity of this membrane, as at the nose, lips, eye-lids, or verge of the anus, is a very constant symptom: the state of the skin, urine, appetite, and bowels, the defect of nutrition, with other symptoms, all lead to the same conclusion. In almost every instance of the disease the symptoms do likewise afford evidence of disorder or disease in the hepatic system. The tendency of affections of these membranes to assume an intermittent or remittent type is a very curious circumstance, and one of which no satisfactory explanation has as yet been proposed. Inflammation of the conjunctiva of the eye and lids possesses occasionally a distinctly remittent character. The symptoms of an intermittent are now and again produced by irritation in the urinary passages. Affections of the mucous membranes often run a very protracted course, and yet leave behind no material change of structure." 317.

In the "bilious disorder" of this country, now so universal among all classes of society, the derangement of function, our author thinks, and with reason, is not restricted to the liver. It takes a wider range, and includes within the sphere of morbid action, those important viscera situated in the vicinity of the biliary organ, and from whence the nutritive particles which supply the body proceed.

We have thus given a very copious account of Dr. Marsh's paper, which is one of the most important and interesting in the volume under review.

### 7.—*A Case of Diseased Heart, with Observations.* By THOMAS CUMING, M.D. &c.

THE subject of this case was a man 38 years of age, of spare habit, and appearing (6th Dec. 1820) slightly emaciated by illness. He stated that about the end of September of the same year, he was seized one day with so severe a pain in the middle of the sternum and in the bend of the left arm, that he was forced to go into a public house, where the symptoms subsided in twenty minutes. While walking homewards he was again seized in a similar manner, but he persevered in walking off the fit. Three days afterwards he had a recurrence of the paroxysm. From this time till our author saw him, he had generally two attacks daily, at 2 o'clock and at 5, attended with precisely the same symptoms, and generally lasting about an hour. In the intervals, as well as in the paroxysms, the patient had strong pulsation of the heart and large arteries, so as to be visible to a considerable distance. On applying the hand to the left side of the chest, the pulsation was felt all over that region, and downwards to below the umbilicus. The face was somewhat bloated, and there had been some œdema of the ancles for a few days—urine scanty and high-coloured—some thirst. Could lie easier on the right side than on the left—subject to flatulence since his illness*—and to an aggravation of a constitutional cough. Before Dr. Cuming saw him he had been twice bled, and blisters had been applied to the chest. He had taken purgative medecine, and draughts composed of digitalis, hyoscyamus, laudanum, and antimonial wine. The bleeding and blistering had relieved his cough, but no impression had been made on the paroxysms or pulsation. Rigid abstinence, quietude, aperients, and blisters were ordered, but no favourable change occurred—on the contrary, from the 7th till the 23d of December, he had seldom less than two, frequently three or four paroxysms throughout the night, and occasionally as many throughout the day.

"The pain of the arm was at times so excruciating as nearly to induce delirium; and whenever this was the case I observed he had but little corresponding pain of the sternum. On some occasions he had slight pain of the arm without any pain of the sternum. During the paroxysms the face was generally pale, and the body bathed in perspiration. Frequent attempts to expel flatus from the stomach generally attended each paroxysm, and he always felt considerably relieved after its expulsion. Although the pulsation was always strong, and communicated a vibratory motion to the bed clothes, it was more violent during the paroxysm. His sufferings during the paroxysm were not aggravated, as in the case of angina pectoris, when he walked about; and when seized with it at night he was always obliged to sit up immediately in bed." 323.

Friction and grasping the arm relieved the pain on one or two occasions; and frictions to the chest generally brought some mitigation of the pain there and in the arm. Opiates failed to relieve the paroxysms.

On the 23d December, some soreness of the gums took place from calomel he had taken combined with ipecacuan for some days previously. His urine increased in consequence, and the œdema diminished. The paroxysms continued as before. From this time, however, till the 1st of January, they were less frequent and severe than hitherto—particularly the pulsation, from which he was sometimes entirely free. On the evening of the last mentioned day, he was seized with a violent fit of coughing, which lasted without intermission till 2 o'clock the following morning, obliging him to sit up in bed all that time. From this hour till 7, he laboured under incessant dyspnœa, with gasping for breath. For several

* Flatulence is a remarkable and a very constant attendant on many organic diseases of the heart, especially on angina pectoris. It is still more inexplicable than the pain in the arms and some other phenomena accompanying this melancholy class of maladies. *Rev.*

nights after this, he was obliged to sit propped up in a chair, on account of a sense of impending suffocation. From this time till the 13th of February he fluctuated, sometimes a little better, sometimes worse. He died on the morning of the last-mentioned day. He was examined in the presence of several eminent medical gentlemen.

"The right side of the chest contained from 6 to 8 oz. of fluid. The right lung was adherent to the parietes at the lower and back part by strong membranous bands. These adhesions, it was, however, evident, were not of recent formation. The lung itself was thickened, and its texture more than usually solid. When cut into, a frothy mucus flowed out. The left side of the chest was apparently healthy. The pericardium was considerably enlarged, and contained about four ounces of fluid. It was not thicker than natural; but on the external surface its vascularity was increased, more particularly towards the left side. The heart was fully double its natural size. The external surface of the left ventricle was highly vascular, and towards the base of the heart presented a granulated appearance. This appearance did not extend to the right ventricle. The coronary arteries were larger in proportion to the size of the heart, but in every respect healthy. The right cavities were increased in size, but otherwise their appearance was natural. The great increase of size was in the left ventricle, whose cavity was so much enlarged, as to admit of the hand being turned round in it with facility. The parietes of the left ventricle were thinner than natural, and the musculi pectinati more projecting.

"The tricuspid and mitral valves were perfectly healthy, as were also the semilunar valves of the pulmonary artery. The valves of the aorta presented a shrivelled appearance; their margins were irregular, thickened, and of a cartilaginous consistence. When stretched to the uttermost, they did not reach within the tenth of an inch of the openings of the coronary artery. The aorta, throughout its entire course, was perfectly healthy, with the exception of a small opaque spot near the heart, which in reality could not be called a morbid appearance." 327.

Nothing worthy of notice appeared in the abdomen, excepting the great distention by flatus of the stomach and bowels.

Our author observes, that the violence of the heart's action, conjoined with a strong, full, vibrating pulse, left no doubt on his mind that the case was "active enlargement of one or both ventricles of the heart." He was, therefore, not a little surprised "to observe a condition of parts the very opposite of that which he had been most confident of finding." When Dr. Cuming has attended more to cardiac diseases, he will find that it is not so easy a matter to distinguish, *by symptoms*, that enlargement of the heart which is attended with thickening of its parietes, and that which is *not* attended with thickening. In extreme cases, indeed, the diagnosis will not be very difficult. For instance, if the cavities are greatly enlarged and the parietes greatly extenuated, we will not have such a state of the pulse as described in the case above; but where the heart is double the natural size, and yet where the parietes are somewhat thinner than natural, we may, and very often will have a preternatural action of the organ, manifested by the pulsations in the chest and in all tangible arteries. Such was the case in Dr. Cuming's patient. We do not look upon the heart as *passively* enlarged by any means—on the contrary, we consider it a case of hypertrophy of the organ, although the parietes were not thickened. The action of the heart too was that of hypertrophy. This case shews us, as we stated in our last volume, page 810, that "the train of phenomena presented in angina pectoris is produced by various and often dissimilar affections of the heart." The patient of Dr. Cuming's evidently laboured under what is commonly denominated angina pectoris. The infarcted state of the lungs was a consequence, no doubt, of the embarrassed action of the left ventricle, on account of the imperfection in the ventriculo-aortic valves.

8.—*A Case of Fatal Hæmorrhage, Six Weeks after Ligature of the Carotid Artery.* By J. W. Cusack, M. D.

A young hypochondriac attempted suicide by inflicting a deep wound in his throat with a knife, on the 26th August, 1820, and was immediately conveyed to Steven's Hospital, where our author saw him a few minutes after admission. He was recovering from a faint, his breathing laborious, and his pulse scarcely perceptible. The wound extended from the sterno-mastoid muscle of the left side, crossing the centre of the thyroid cartilage, to the angle of the jaw on the right side. The thyroid cartilage was almost completely cut through. The sheaths of the large vessels on both sides of the neck were exposed, and the pulsations of the carotids visible. The parts were dressed superficially, but at 7 in the evening, blood gushed from the right side of the throat, and the patient fainted before the blood could be restrained. Our author had no doubt that the carotid or one of the large branches was wounded. A ligature therefore was applied to the trunk of the vessel, and also to the thyroid artery of the opposite side, which was exposed and had its coat injured. On the 24th, the patient was in a feverish and bad state, the wound looking unhealthy, and a slight hæmorrhage having taken place. Bleeding and digitalis removed these symptoms, and the patient appeared to be doing well till the 16th of September, when he became harrassed with a pectoral affection, which, however, subsided before the 1st of October. By the end of the second week in this month the wound had closed, except a small opening, about three lines in diameter, in the thyroid cartilage. On the 15th of October he complained of pain, and a slight fulness about the mastoid muscle—the wound looked unhealthy, and fever was present. Saline purgatives —poultices—quietude—abstinence. At four o'clock on the 15th blood suddenly gushed from the wound, and was partially restrained by pressure. When Dr. Cusack saw him it was evident that the carotid had burst, and it was determined, in consultation, that the vessel should be secured nearer to the sternum. This was rendered difficult by the close adhesions which had consolidated the cellular texture. After exposing the sheath of the vessels about half an inch above the origin of the subclavian artery, our author made an opening into it; but, after passing the ligature, and tying the knot, his efforts were fruitless, as on removing the pressure the hæmorrhage recurred with undiminished violence, and the patient's existence was soon terminated.

" *Dissection.* After the integuments were removed from the course of the right carotid, the sac of a small abscess was exposed, occupying the situation of the first ligature. Its lateral diameter did not exceed one inch, extending from the opening into the larynx to the right side of the sheath of the vessels. Its extent from above downwards was less. The lower extremity of the carotid was visible, it was distant from the upper about half an inch, both were pervious, and the edges of their orifices were ulcerated and irregular. In prosecuting the dissection, the parts were found closely consolidated. The sheath adhered so closely to the vessels and the cellular texture surrounding it, that no infiltration of blood could have taken place. Having removed the larynx and trachea, with the vessels on each side, the injured carotid was slit open; its coats were found considerably to exceed in thickness those of the artery on the left side." 342.

### 9.—*A Case of Traumatic Tetanus, successfully treated, by Tobacco; with Observations.* By James O'Beirne, M.D. Surgeon Extraordinary to the King, &c.

Dr. O'Beirne observes that "much of the capricious and *unmanageable* character of tetanus may, with strict justice, be imputed to the want of any philosophical views of either its seat or its causes." As tetanus has been treated upon every possible hypothesis as to its etiology and pathology with nearly the same success—or rather want of success, so, we fear, that it would still maintain its *unmanageable* character, were its seat and causes ever so well known. By this observation we do not wish to check inquiry, but to moderate anticipations in respect to therapeutics. We are convinced that, in many diseases, the extension of our investigations will be the abridgment of our hopes of cure. Knowledge will shew us good and evil. It will shew us what we *can* do —and, alas! what we *cannot* do, which will be a great advantage after all, though sufficiently mortifying to human pride. It is highly probable that there is, even now, as much mischief done by ignorance, as benefit conferred by skill, in the unlimited field of medicine! The progress of science will have a constant tendency to check this evil, alike distressing to humanity and degrading to the profession.

To return to tetanus. Dr. O'Beirne enters pretty fully into the various hypotheses and conjectures which have been entertained in ancient and modern times respecting tetanus. It was natural enough, on seeing the voluntary muscles so much affected, and the sensorial functions so unimpaired, to place the seat of the disease in the medulla spinalis, the source of the nerves supplying the said muscles. Galen, therefore, gave this opinion, and has been followed by Fernelius, Willis, Hoffman, Burserius, and several others, whose examinations of the spinal brain gave an importance to the theory which a mere hypothesis could never have attained. This theory, however, like many others, sank into neglect or was totally forgotten till within these few years, when anatomical examinations of the spine have again attracted the notice of the profession. Some physiological facts were calculated to awaken our attention to this subject:—Thus Walther produced artificial tetanus by plunging a very slender stiletto into the spinal marrow of a frog, after the head had been severed from the trunk. The same phenomenon was observed to ensue on pressure being made upon the cerebral extremity of the medulla spinalis of such acephalous fœtuses, as, for a short time, survived birth. It was reserved, however, for Le Gallois, Philip, Brodie, and some others, to produce, by direct experiments on the nervous system, results both surprising and unexpected, confirming the experiment of Walther, and exhibiting numerous other curious and important phenomena. Dr. Sanders, of Edinburgh, has long been investigating this subject, and has already laid many important facts before the public. It is well known that this gentleman states his having almost invariably found appearances of inflammation or vascular turgescence in either the tuber annulare, medulla oblongata, or medulla spinalis, of tetanic subjects. In trismus these appearances were confined, in many instances, to the tuber annulare and medulla oblongata, while the spinal marrow remained healthy. Dr. Reid, of Dublin, has published some curious circumstances tending to elucidate the same subject. Although other investigators have not been able entirely to confirm these views by anatomical proofs, yet there can be no rational doubt that the spine is the fons et origo of the formidable disease under consideration, and that we are in the right path of investigation, though, probably, a long way from the end of our journey.

In what way the reputed causes of tetanus produce the morbid condition of the spine and irregular function of the nerves arising from it, is no very easy matter to explain. Our author thinks it incumbent on him to attempt something like a ratio symptomatum in this case, though with much modesty. As tetanus follows so small a number of wounds, comparatively, Dr. O'Beirne thinks it reasonable to conclude that some predisposition or peculiarity must exist in the constitution of the individual attacked, and if this could be ascertained it would be a great object to remove it. He divides the causes then into exciting and predisposing. Under the first class must be reckoned wounds, the action of cold, atmospheric influence, passions of the mind. The class of predisposing causes is large, and many of its items have been glanced at by our author.

Heurteloup mentions a case where the intestines were blocked up with cherry-stones—and many cases of cholera end in tetanus. Mr. Abernethy found the chylopoietic functions out of order, and Dr. M'Arthur has described a "peculiarly offensive smelling yellow matter" pervading the intestines of tetanic patients. Constipation has been often observed to precede this disease. Worms have been very often found in the intestines of those who died of tetanus, and Dr. O'Beirne thinks they are more frequently the cause of this disease than is generally supposed. During life the constipation prevents their discharge, and after death it is but too usual to neglect laying open the whole length of the intestinal canal. To this it may be added that, when worms do exist, they are generally crowded into one spot, and thus may easily elude detection, unless a careful examination be made. As the predisposing causes have been almost exclusively traced to the organs subservient to digestion, our author is led to look upon the splanchnic system as the medium of communication between these organs and the spine, "from which, by a reflected operation, the original sources become themselves excited in their turn." The comparative success which has, of late years, attended the purgative plan of treatment, affords, our author thinks, strong confirmation of the doctrine in question. He gives us the melancholy information, that out of about 200 cases of traumatic tetanus which he witnessed in the Peninsular army, not one recovered. The antispasmodic plan was more employed than the purgative, and he cannot recollect that the spine was ever examined. If our author's views of the etiology of tetanus then are admitted, they necessarily lead, he thinks, to the following indications of cure, viz. 1st. To have recourse to the most powerful remedies for overcoming constipation and destroying worms. 2dly. To employ those which shew the most decided empire over the nervous system. Of all modes of treatment, observes Dr. O'B. "the combination of the purgative and antispasmodic plans has been the most generally successful." Under such circumstances our author thinks it strange that tobacco, which concentrates within itself such various properties, and has shewn such marked influence over diseases of the same class, should not have been frequently and fairly tried. The earliest instance of its mention or employment in tetanus, was by the late Mr. Royston, in his Medical History of Tobacco, in which he states that Dr. Edmund Gardiner published a little work in the beginning of the 17th century, entitled the "Triall of Tobacco," asserting that "the suffumigation of tobacco being taken is a good remedy for the starkeness or stiffness of the neck called tetanus," &c. Baron Larrey applied poultices of tobacco leaves to the wounds of many of the soldiers who were affected in Egypt with this disease, but without any alleviation of the symptoms. Mr. Thomas Duncan, of Grenada, relates a case cured by tobacco-smoke; and on the authority of the same gentleman it appears that Mr. Harris, Surgeon of His Majesty's ship Magnificent, after Rodney's victory in the West Indies, recovered a case of traumatic tetanus by the fumes of tobacco blown up the nostrils. But it is time to give an account of the case which led to these observations and researches.

*Case.* J. Flood, aged 13, a healthy strong boy, had his feet miserably torn by approaching too near some machinery, on the 16th of August. He was immediately conveyed to the Charitable Infirmary, and had not been exposed to cold or wet. Emollient poultices were applied, and the bowels opened by sulphate of soda. In a week the wound exhibited a sloughy appearance, and the boy was slightly delirious. On the 25th of August, he complained of stiffness of the neck, and pain in turning the head—bowels constipated. Next day the rigidity of the neck was quite perceptible. 27th. Complete trismus—slight opisthotonos—bowels obstinately confined. On the 30th, all the symptoms being aggravated, it was determined to use the tobacco-glyster, (a scruple to the half pint,) and to repeat it the same night. The injections could not be forced up during the tetanic paroxysms, so powerful was the constriction of the rectum. In the intervals, however, they were passed up with ease. Each enema was retained about two minutes, and caused nausea, vomiting, copious perspiration about the head and chest, tendency to deliquium, and sense of great heat through the intestinal canal. The general symptoms were rather worse on the 31st. The injections to be twice repeated this day again. Sept. 1st. One of the injections yesterday brought down some black indurated fæces, and a dead lumbricus about one foot in length. Spasms less frequent to-day—neck less rigid. 2d. Had two injections yesterday. Continues better—deglutition more easy. These enemas were continued with more or less frequency till the 17th of September. They generally opened the bowels copiously, and relieved the tetanic spasms. By the 8th of October the spasms had disappeared, and he completely recovered.

Dr. O'Beirne has filled many pages with reflections on this case—indeed he is rather too prolix; for a single case can do no more than authorize further trials, without exciting any thing like sanguine hopes, or forming any basis for general conclusions. It appears to us that the principal indications of a useful kind, which the tobacco enema is calculated to fulfil, is the evacuation of the bowels. From the state of the sphincter and of the rectum, we may fairly conclude, that the whole line of the intestinal canal participates in the tetanic spasms, and therefore the tobacco, cautiously administered, and properly proportioned, may be a very useful mean of overcoming this rigidity, and keeping up a soluble state of the bowels, so highly desirable in this formidable disease. It is always to be borne in mind, however, that tobacco is very uncertain in its effects on different individuals, and even on the same individual, in different circumstances. The dose should therefore be small at first, and the effects carefully watched.

### 10—*An Account of two Cases in which Bronchotomy was performed.* By Richard Burgess, M.D.

Our readers will remember some cases of violent inflammation about the larynx produced by children swallowing hot water out of the spouts of tea-kettles, as described in the Medico-Chirurgical Transactions, and analyzed in this Journal. The cases in the work before us are of a similar nature. In the first case the mouth, tongue, and fauces were severely scalded, so as to prevent deglutition, and considerably impede respiration. On looking into the mouth two hours after the accident happened, "it appeared as if a large piece of raw flesh had been forced into the fauces, and had completely filled up the passage." Respiration was very difficult, and becoming more and more laborious—in fact, the child appeared to him to have but a short time to live. Bronchotomy was therefore performed with some difficulty, and relief was instantaneously obtained. The usual treatment for inflammation was pursued. About the 8th day deglutition was practicable, and on the 10th, air could be breathed by the natural passage.

The second case was similar; but our author did not see the child till eight hours after the accident. "She appeared lifeless;" Mr. Burgess opened the wind-pipe, and the child instantly revived. She died, however, in about twelve hours after the operation, in consequence of the attendants neglecting to keep the wound free from the mucus which collected within it.

11.—*Case of Strangulated Hernia, occasioned by a double Stricture in the Sac, of an Hour-glass Shape.* By Richard Carmichael, M.R.I.A. Surgeon of the Richmond Hospital, &c.

It does not follow that a case is extremely rare because it has never been described. Many things happen, and are known to individuals, but never recorded. Very many things daily happen, of an extraordinary nature, which are neither observed nor recorded. What has happened once, therefore, we may reasonably calculate upon happening again, where observation is directed to the point. We are inclined to think that the case recorded by Mr. Carmichael has not unfrequently been the unsuspected cause of failure in operations for hernia:—and if this be the fact, the circumstance about to be stated deserves to be treasured in the memory of the surgeon.

On the 6th of December, 1821, a middle aged man was brought into the Richmond Hospital with strangulated hernia, stating that the symptoms had come on the preceding evening. Every attempt to reduce the protruded parts by the taxis proved unavailing; but the symptoms not being urgent, the operation was deferred till next morning, and a further trial given to blood-letting, purgative pills, warm bath, and tobacco injection. These also failing, Mr. C. in consultation with Mr. Todd, proceeded to the operation. He first endeavoured to return the protruded parts (a scrotal hernia of the left side) without exposing them, but could not succeed this way in returning the hernia. The sac was therefore opened, and a knuckle of intestine disclosed of a livid colour, but not mortified, and a large volume of omentum, some of which firmly adhered to the lower part of the sac. The stricture which was formed by the neck of the sack gave way to the pressure of the finger, or was rather torn through by the nail, and the intestine was, in consequence, reduced with much ease. But greater difficulty was experienced in returning the omentum, the major part of which was, however, reduced.

The patient being put to bed, and an enema exhibited, no motion followed. In the evening he was much worse. Numerous leeches were applied to the abdomen, and a large bleeding taken from the arm. Great gastric irritability. On the morning of the 8th he died.

"On examination—The contents of the abdomen were found free from inflammation; but neither the strangulated intestine nor omentum could be discovered. It was evident that they had not been returned into the cavity, although when operating I had pushed them as far upwards as my finger could reach into what I conceived was the cavity of the abdomen. Further dissection explained these perplexing and extraordinary circumstances. It was found that between the internal and external rings, the sac had expanded itself into a large cyst, which extended into the bason of the pelvis on the one side, and into the hollow of the ilium on the other, lying between the muscles, being covered anteriorly by the external oblique, and separated from the cavity of the abdomen, by the peritoneum, the fascia transversalis, the transverse, and internal oblique muscles. In this cyst the inflamed intestines and the omentum were discovered, and found to be completely strangulated by the internal ring. I do not recollect any systematic writer on hernia who has noticed this hour-glass sac and double stricture; but having been ascertained in one instance, no doubt it has often occurred, and occasioned the failure of the operation, and as a fact of some practical importance deserves to be made generally known.

"Even had I been aware of this unlooked-for formation of the sac, I could not have removed the stricture of the internal ring in the usual way, as it lay far beyond the reach of the finger from the external ring; I should therefore have been obliged either to cut down directly upon the former, or else have continued the incision from the one to the other. The protruded parts thus being in this case subjected to two strictures, there can be little doubt but that of the internal ring was the primary one, and the greater of the two, and that the strangulation was probably caused by the descent of an additional portion of intestine or omentum; while that caused by the external ring was probably but secondary, and produced by the swollen state of the intestine from the stricture above." 386.

The peculiar formation above described, Mr. Carmichael thinks, arose from a careless or injudicious use of the truss, which is generally applied by the patient so near to the os pubis, as to be only capable of closing the external ring, while the herniary tumour is allowed to remain between the two rings—a space which, as is seen in the case related, may be gradually extended from the increasing size of the herniary mass, while it is prevented from passing downwards by the application of the truss at the external ring. This case, therefore, should afford a warning both to surgeon and patient, respecting the application of the truss; and shews the necessity of placing the pad exactly over the site of the internal ring. From the experience of this case, our author would be extremely unwilling, on future occasions, to leave any portion of omentum unreduced, as its presence at the ring must prevent, in a great degree, the surgeon from ascertaining with proper accuracy whether any farther stricture remains undivided.

12.—*A Case of Dislocation of the Hip-Joint, with the Manner of its Reduction, and the Appearances on Dissection.* By James Scott, M.D. Surgeon to the County Infirmary of Armagh.

The recent morbid anatomy of dislocations must be rather defective, from the rarity of these accidents proving fatal, and consequently the rarity of anatomical investigations. There are not many cases on record of the post-mortem appearances in luxation of the hip-joint, and therefore the following particulars are deserving of universal diffusion. The patient was working in a gravel-pit when a bank fell in upon him. In six hours after this accident he was seen by Mr. Scott. The thigh was shortened and deformed, the muscles flaccid, the knee and toes turned inwards. Every attempt at rotation outwards caused extreme pain in the groin, at the hip, and in the course of the sciatic nerve. The description of the reduction we need not notice, as our late numbers have contained enough on that subject. Shortly after the poor man was put to bed, it was discovered that a serious injury had been received in the abdomen, which caused his death in 36 hours after the accident.

" On dissecting down to the hip-joint, an extensive extravasation of blood presented itself in the cutaneous cellular substance, covering the trochanter major, and also beneath the fascia lata of the thigh, extending several inches above and below the trochanter. The gluteus magnus being raised from its origin, a considerable extravasation was found in the loose cellular tissue under the gluteus medius: a cavity capable of containing a pullet's egg was also brought into view. This cavity was situated directly where the great ischiatic nerve passes under the pyriform muscle; it contained fluid blood; its boundaries were the pyriformis above, the sciatic nerve before,

(supposing the body upright,) the trochanter major, and insertion of the gluteus medius external and posterior, the gluteus maximus posterior. Here the displaced head of the femur had been lodged. The fleshy substance of the gemini and quadratus muscles was found torn across. The pyriformis and obturator internus were perfect. The extravasated blood followed the course of the sciatic nerve deep into the thigh. There was also extravasation between the gluteus medius and minimus muscles.

"The internal and upper part of the capsular ligament of the joint was ruptured; the external portion remained unbroken. On turning the head of the bone out of its socket, the ligamentum teres was found to have been torn from its insertion into the dimple of the head of the thigh bone. The brim of the acetabulum at its upper part was fractured to the extent of about an inch; the fractured portion lay loose and nearly unconnected. A fracture traversed the acetabulum in the direction of the junction of the ileum (ilium) and ischium." 393.

13.—*An Account of a Dissection of the Hip-Joint after recent Luxation. With Observations on the Dislocation of the Femur upwards and backwards.* By CHARLES H. TODD, one of the Senior Surgeons to the Richmond Surgical Hospital, &c.

IN the summer of 1818 a robust young man fell from the window of a second floor into a flagged area, by which he fractured the cranium, and dislocated his left thigh upwards and backwards. The dislocation was reduced without difficulty, but an extensive extravasation of blood in the brain put a period to the patient's life in 24 hours. On the following day the examination was made of the injured joint and parts contiguous.

"On raising the gluteus maximus, a large cavity filled with coagulated blood was found between that muscle and the posterior part of the gluteus medius. This was the situation which had been occupied by the dislocated extremity of the femur. The gluteus medius and minimus were uninjured. The pyriformis, gemini, obturatores, and quadratus, were completely torn across. Some fibres of the pectinalis were also torn. The iliacus, psoas, and adductors were uninjured. The orbicular ligament was entire at the superior and anterior part only, and it was irregularly lacerated throughout the remainder of its extent. The interarticular ligament was torn out of the depression on the head of the femur, as in Doctor Scott's case, its attachment to the acetabulum remaining perfect. The bones had not sustained any injury." 396.

We are unable to give Mr. Tood's reflections and criticisms on Boyer, Richerand, Sir A. Cooper, &c. and therefore having stated the fact, we refer to the work itself for the comments.

14.—*On Diseases of the Lachrymal Gland.* By Charles H. Todd, &c.

Our author observes, that although this gland is not so frequently affected as its situation, connexions, and sensibility would lead us to expect, yet it is more often drawn into disease than most writers allow.

The lachrymal gland is subject to acute inflammation, sometimes idiopathic, but in the greater number of cases, succeeding to conjunctival inflammation or some other form of ophthalmia.

"The symptoms of acute inflammation of the lachrymal gland are, intense pain in the orbit, but particularly under the temporal extremity of the eye-brow, extending to the temple and cheek, backwards into the orbit, and even into the cranium; defective or profuse lachrymation; when the latter, which is most generally the case, the patient complains of the tears being hot and acrid, and in a few days the edges of the eyelids and the cheeks become excoriated to some extent; both palpebræ, but particularly the superior, are swollen, red and tense; inflammation extends to the conjunctiva and other membranes of the eye, and thus a severe and obstinate form of ophthalmia is produced. More commonly, however, inflammation originates in the conjunctiva, and is thence propagated to the gland.

"When the inflammation of this gland is at its height, there are much symptomatic fever and restlessness; flushing of the face, particularly at the affected side; acute pains darting through the orbit and head; and, as it is stated, more or less delirium, with strabismus, impaired vision, and protrusion of the globe proportionate to the size of the swelling." 410.

The remedies are, of course, general and local blood-letting, warm fomentations, mercurial and saline purgatives, antimonials.

*Chronic Inflammation* of this gland is a disease, Mr. Todd observes, almost entirely confined to the early periods of life, and probably dependent on a scrofulous disposition. In this affection there is an obvious enlargement of the gland, with an occasional œdematous tumefaction of the upper eye-lid, the patient complaining of an inconvenient sensation of fulness, rather than pain, above the globe, and an inability to move that side as freely as the other. This produces strabismus, and double or indistinct vision.

"In this disease the best effects are obtained from the application of a few leeches to the neighbourhood of the gland, at as early a period as possible; from a succession of small blisters to the forehead, temple, and behind the ear; from small doses of calomel, blue pill, or some other mild preparation of mercury, with the occasional use of saline laxatives; and from such medicines as an imperfect performance of the digestive functions may indicate. Local applications, except where the conjunctiva or eye lids are inflamed, or the cornea ulcerated, are unnecessary; and even in such cases the benefit derived from them will be small and of short duration, unless our exertions to restore the gland to an healthy state be effectual." 412.

Besides acute and chronic inflammation, the lachrymal gland is subject to enlargements, (scrophulous) abscesses, and scirrhus; for cases of which, we refer to the present paper.

### 15.—*Observations on the Treatment of Diabetes Mellitus.* By Henry Marsh, A. B. M. D. &c.

Dr. Marsh thinks that, since the publication of Dr. Rollo, and the investigations that followed, diabetes mellitus has lost somewhat of its hopeless and intractable character. Very small, we think, has been the loss; and as to the animal food treatment, the efficacy of which was, at first, scarcely doubted, it will be found, as Dr. Marsh observes, "greatly to disappoint the expectations of those who are disposed to place much reliance upon its virtues." There can be no doubt, indeed, that an exclusively animal diet may alter considerably the sensible properties of the urine, and materially diminish its quantity, but even with the few who can endure such restriction, little will be done towards the removal of the disease. A partial adoption of this regimen will, however, be useful, and merit is undoubtedly due to Dr. Rollo for his discovery respecting the change produced in the saccharine urine by a total abstinence from vegetable matter.

Amongst the remedies which have been employed, opium in large doses has ranked high. It checks the flow of urine, and deprives it of many of its morbid qualities. Its effects, however, are of a transient nature, and when the medicine is withdrawn the complaint recurs. A permanent cure of the diabetes mellitus has rarely been accomplished, and a perfectly successful mode of treatment yet remains to be discovered.

A well-marked case of this disease was lately presented to our author's notice, and he naturally searched for all the information he could collect from books. While employed in these researches his attention was particularly arrested by the following important considerations.

"First, in many of the cases whose histories are recorded, the earliest disturbance in the general health could distinctly be traced to some cause acting upon the skin, and producing derangement of its functions. Secondly, *every* case of the Diabetes Mellitus is accompanied with a peculiarly morbid condition of the skin. In truth, I know not any disease, in which this symptom is so uniform and so remarkable. Thirdly, none of the remedies employed produced the slightest beneficial effect, until the skin began to relax, and a sweat to appear on the surface.*

"These considerations led me to turn my attention more particularly to the state of the skin, and suggested the probability of advantage arising from the application of *vapour* to the whole surface of the body. The vapour bath was employed. The impression made upon the disease by the frequent use of this remedy surpassed my expectations. Its salutary effects, in giving a new action to the skin, were immediately perceptible, The perspiration having been afterwards maintained by warm clothing and continued bodily exercise, the patient daily improved in health; and at length quitted the hospital, under the conviction of his disease being wholly removed." 434.

Our author, after making several useful remarks on certain cases in Dr. Rollo's and Dr. Latham's publications, proceeds to a detail of the principal facts connected with his own case.

The patient was a shoe-maker, 20 years of age, sallow complexion, great emaciation, full and prominent veins, skin a dingy yellow, permanently acrid, and glued apparently to the subjacent muscles; gums ulcerated; a small unhealthy ulcer on the right cheek; epigastrium tumid; tongue florid at margin and point, and covered in other parts with a thin whitish secretion; extreme listlessness, languor, debility; a sensation of weakness referred to the knees; dimness of vision;

---

"* See two cases of Diabetes treated by opium. *Transactions of the London College of Physicians*, vol. iv. In Case I, it is stated, p. 198, that 'the opium produced considerable perspiration, and on the following morning the urine was no longer sweet. The patient had felt great relief from languor since the opium had been resumed.' In Case II. p. 208, it is observed that, 'during the period in which the dose of opium had amounted to ten grains (taken four times in the day) the patient had perspired profusely, had been sleepy and giddy, but had suffered no other inconvenience: the urine had a natural appearance and odour, and yielded a very inconsiderable extract.' A case is recorded by Dr. Darwin, in which opium produced salutary effects, at a time when it caused the patient so to sweat, 'that large drops stood on his face, and all over him.' Another case is mentioned by the same author, in which a course of astringent and tonic medicines did not in the least benefit the patient. Opium was at length given; it excited profuse perspiration, and great relief ensued. Emetics have been useful, so far as they have been effectual in determining to the surface. The Hepatized Ammonia has also had the effect of promoting perspiration. Antimonials, when effectual in exciting diaphoresis, have likewise been found useful. Blood-letting and the warm bath are valuable remedies, and their utility will be found proportionate to the power they possess of re-establishing the functions of the skin." See Watt on Diabetes, pages 27, 28, 29, 36, 38, 151, 152, 155."

disturbed sleep; sensation of creeping coldness over the surface; appetite inordinate; thirst unquenchable; mouth clammy; digestion rapidly performed, with a craving sensation recurring soon after food is taken; costiveness; constant desire to make water, which is increased during the night. From 20 to 22 pints of urine are passed ordinarily in the 24 hours—the bubbles remaining on its surface, but limpid and almost colourless, its smell peculiar, and not easily to be described; taste very sweet. When evaporated, an abundant extract resembling coarse brown sugar remained—pulse 86, and throbbing—neither cough nor dyspnœa. Such were the symptoms on the patient's admission into hospital towards the end of December. He stated, that in the beginning of the preceding November, while crossing from Liverpool to Dublin, then in perfect health, he encountered a violent storm, and was exposed to much wet, cold, and hunger, during four days. After quitting the vessel he felt himself constantly chilly, to which intense thirst succeeded, and he was perpetually swallowing large draughts of cold water. His skin he described as very dry, with a total want of perspiration, dimness of vision, and costive bowels. Languor and debility increased notwithstanding the large supplies of food; and at length he was obliged to abandon his ordinary occupation, and seek relief in an hospital.

On the 1st February, venesection to ten ounces, the serum being milky, the crassamentum firm. The symptoms were unaffected by the bleeding. From this time, on to the second of March, mercury was used externally and internally, in large quantities, without fetor of the breath, or increase of saliva indicating mercurial influence. He lost ground during the use of this medicine, and at length was unable to leave his bed through weakness. On the 2d March, the vapour bath was tried, and repeated on the 9th, 12th, and 26th, the symptoms still continuing unabated, but with perhaps some slight accession of strength. From the 2d to the 27th April, all treatment, except daily purgation, was laid aside. The sweet urine often exceeded 24 pounds in the 24 hours. On the last-mentioned date he was again placed in the vapour bath, half an ounce of the tincture of opium being mixed with the water. After remaining 20 minutes in the bath, syncope supervened, after which he became feverish and hot. At length the skin opened, and the whole surface of the body became covered with sweat. He felt immediately relieved, and on the following day was much better. On the 4th May he was again put into the vapour bath with the tincture of opium as before. There was no tendency to faintness; but a copious perspiration followed, and was succeeded by a sound and refreshing sleep. On the 10th May, a note was made that his strength was improving daily—that he felt much less languid—skin soft and perspiring—pulse 88—appetite less craving—improved sleep—increase of flesh—gums still ulcerated. 18th May, urine almost colourless and very sweet, amounting during the preceding 24 hours to 24 pints—thirst urgent. May 22d, a temporary diarrhœa; during the increased action of the bowels, the urine assumed an amber colour, and acquired a urinous odour, being much diminished in quantity and saccharine quality—tongue cleaner—fæces yeasty—no perspiration—skin itchy. May 30th, urine still amounts to 21 pounds—pulse 100—no perspiration. Had been ordered for sometime past an exclusively animal food diet, with lime water and milk for drink; but he secretly devoured whatever vegetable food he could procure. June 3d, a remarkable change in the symptoms—only 8 pints of urine during the last 24 hours, the perspiration being copious—great accession of strength, and diminution of thirst and appetite. The perspiration was produced by laborious exercise, the body being enveloped in flannel, and the weather being warm. From this period till the beginning of July, medicines of every kind, were left off—he worked hard every day—was warmly clad, and sweated much. His food was principally vegetable—he daily gathered strength and weight—enjoyed sound undisturbed sleep, and felt himself so far relieved that he was resolved to return home and resume his original avocations. A little persuasion induced him to wait some time longer under medical superintendance. But we cannot pursue the daily routine of treatment, which varied according to circumstances. In the month of September he quitted the hospital.

"I saw him on the first of January, 1822. There was not any return of the symptoms; the skin retained its natural softened feel; he weighed eight stone five pounds; his pulse was moderate: the expression of his countenance very much improved: though not exempt from thirst, it gave him no annoyance: his tongue was whitish; he felt strong, and alive to every enjoyment; he worked at his trade as a shoemaker, from an early hour in the morning, with little intermission, until late at night; his diet consisted of bread, butter, occasionally meat, fish, potatoes and gruel: the gums were very slightly ulcerated; the bowels, without medicine, were daily evacuated; the general quantity of urine, in the course of a day and night, varied from six to seven pounds; taste salt, slightly saccharine; colour still paler than natural." 449.

Such are the leading facts belonging to this case. Although we are far from thinking the patient perfectly cured, we grant that the case exhibits, in a striktng point of view, the powerful effects of copious perspiration, whether produced by the vapour bath or muscular action, in controlling the symptoms of diabetes. Diaphoretics are, of course, no new remedy in this disease; but they have generally been exhibited internally, and have been ineffectual in rousing the action of the skin. The etiology of the disease in this case, seemed to point to the vapour bath as a powerful counteractor of the impression made by wet and cold on board the vessel. But having once relaxed the skin, and brought it into a perspirable state, it is of the greatest importance to keep up the said condition of surface, and this seems best done by exercise and clothing. It would appear necessary that the diabetic patient should never allow a day to pass without a certain quantum of exercise, if the strength will at all permit. In the upper classes of society, horse exercise is a valuable remedy, as it excites perspiration without inducing fatigue. To exercise the patient will generally be very averse at first; but experience of its good effects will soon render it agreeable. The same cannot be said of the other remedies, as opium, animal diet, &c. In the case above sketched out, we omitted to state that a very considerable disgust was raised by the animal food—an event not very rare where such diet is exclusively used. We agree, therefore, with Dr. Marsh, that the cure of a disease so generally fatal as diabetes, should never be trusted to a single remedy. A regular and systematic plan of treatment should be entered on, while the exciting and maintaining a general diaphoresis should be considered an indispensible part thereof. For many suggestions and judicious observations on diabetes we must refer to Dr. Marsh's paper, which is creditable to his judgment and research.

The remaining article in this volume, is an operation for axillary aneurism, by Mr. Todd, which we have fully detailed in a former number of this series.

We hope and trust that every year will present us with a new volume of these Hospital Reports, so honorable to the professional character of the sister island.

